# The Medical Profession

**Published:** 1884-03

**Authors:** 


### Editorial.

### The Medical Profession.

> "I know him to be of worth,
> And of worthy estimation."
>
> —*Shakespeare.*

The estimate which a man makes of himself is often the estimate at which he is held by his acquaintances, and especially is this true, if the estimate so far from being an index of vanity and conceit, represents simply a manly independence, and that self-respect which is founded on conscious integrity and a fair and just estimate of worth. As with an individual, so with a profession.

In the medical world the temptation "to crook the pregnant hinges of the knee, where thrift may follow fawning," seems many times irresistible. That in individual cases pecuniary profit may occasionally accompany this practice will not be denied. But in these instances pecuniary gain is moral loss; and what is even worse, this unsanctioned and miscalled gain of the individual is the loss and degradation of the profession, of which such individuals are dishonorable members.

No impartial judge will deny that over-crowding a profession necessarily increases this temptation, in that it morbidly intensifies the strife for existence; and unhappy is it that the "survival of the fittest" can many times be here only admitted in a low sense, as meaning the survival of those most fit for strategems and spoils.

That the medical colleges are, in great measure, responsible for this demoralized state, has many times been clearly pointed out. Their greed and want of principle are peculiarly their own shame; and yet, in some measure, must the whole profession, of which the various college faculties form a prominent part, suffer discredit and the other inevitable consequences of prestige and reputation sacrificed to overweening selfishness. The profession of this city has had, during the past year, proof of the low estimate at which its character and services are held. At least two of the members of the staffs of our most prominent hospitals, after a working lifetime of most faithful services, have received as a reward summary and uncomplimentary dismissal, and this, as we believe, in the one case as a matter of rivalry, and in the other of repaying the incivility, in accordance with the well-known principle that two wrongs make a right. These two physicians, capable and faithful in the highest degree, honored and esteemed by the profession, after years upon years of toil and thought devoted to institutions towards which, from long and intimate association, they had learned to feel a paternal and loving interest, are become the victims of that disgraceful estimate at which the profession and its services are held by a shrewd and captious public.

In the face of this, what can be more humiliating than the thought, that the medical profession, having its own making entrusted to its care, should thus, voluntarily, from apathy or greed, have suffered itself to sink so low.

Utopian as it may appear, we hold it the duty of a physician to refuse preferment under circumstances such as these, where the acceptance involves such gross injustice to a fellow-worker.

To this position of manly independence and self-respect, must the profession rise before it can command or be entitled to the respect of a critical and prejudiced public As a means to this end we regard the State Faculty Bill, and we make the prediction that eventually this honorable standard of medical ethics, compared with which the question of the new code is of the most trivial importance, will be established. Then, and then only, will it be truly said of the medical man: "I know him to be of worth and of worthy estimation."

